# Supplementary material for: Improving personalized tumor growth predictions using a Bayesian combination of mechanistic modeling and machine learning
Source: Commun Med (Lond). 2021 Jul 29;1:19. doi: 10.1038/s43856-021-00020-4 (PMC9053281; doi:10.1038/s43856-021-00020-4)
Supplement: Supplementary file 1 — Peer Review File. [file 43856_2021_20_MOESM1_ESM.pdf]

This manuscript has been previously reviewed at another Nature Research journal. This document only contains reviewer comments and rebuttal letters for versions considered at Communications Medicine.

REVIEWERS' COMMENTS:

Reviewer #1 (Remarks to the Author):

I have previously reviewed this manuscript and I don't have any further comments. The manuscript has been improved drastically from previous review, and I strongly recommend acceptance.
